# Endovascular Treatment for Basilar Artery Occlusion

**DOI:** 10.3390/jcm13144153

**Published:** 2024-07-16

**Authors:** Devansh Gupta, Lucio D’Anna, Piers Klein, Robert Araujo-Contreras, Artem Kaliaev, Mohamad Abdalkader, Wei Hu, Thanh N. Nguyen

**Affiliations:** 1 Smt. Kashibai Navale Medical College and General Hospital, Pune 411041, India; devgupta.neuro@gmail.com; 2 Imperial College NHS Trust, London W2 1NY, UK; l.danna@imperial.ac.uk; 3 Department of Neurology, Boston Medical Center, Boston University Chobanian and Avedisian School of Medicine, Boston, MA 02118, USA; piers.klein@bmc.org (P.K.); robert.araujocontreras@bmc.org (R.A.-C.); 4 Department of Radiology, Boston Medical Center, Boston University Chobanian and Avedisian School of Medicine, Boston, MA 02118, USA; akaliaev@bu.edu (A.K.); mohamad.abdalkader@bmc.org (M.A.); 5 The First Affiliated Hospital of University of Science and Technology of China, Hefei 230026, China; andinghu@ustc.edu.cn

**Keywords:** basilar artery occlusion, endovascular therapy, acute ischemic stroke, large vessel occlusion

## Abstract

Basilar artery occlusion (BAO) is a neurological emergency associated with a high risk for adverse outcomes. This review provides evidence on the therapeutic efficacy of intravenous thrombolysis (IVT) and endovascular therapy (EVT) in the treatment of BAO. Historically considered the primary intervention for acute ischemic stroke, IVT has been progressively combined with EVT, which has emerged from recent studies demonstrating clinical benefits, notably in patients presenting with severe stroke. Several randomised controlled trials have shown that EVT improves patient outcomes in select clinical contexts. Future research directions could address therapeutic treatment thresholds, combination strategies, and long-term outcomes.

## 1. Introduction

Stroke is a significant public health concern globally due to its high prevalence, mortality, and long-term disability implications. Despite advancements in treatment and prevention, stroke remains the third leading cause of long-term disability and the second leading cause of mortality worldwide [1].

Basilar artery occlusion (BAO), a severe form of stroke, presents unique challenges in terms of its management [2]. BAO can be a devastating neurological condition, with disability or death occurring in up to 70% of patients [3,4]. BAO is relatively uncommon, accounting for only 1% of all ischemic strokes. In accordance with its severity, BAO makes up a larger proportion of endovascular thrombectomy cases, approximately 6 to 8% [5]. In a prospective international study of individuals with BAO, the overall mortality rate was 36%, and only 32% achieved positive clinical outcomes, defined as a modified Rankin scale (mRS) score of 0 to 3 at one month [3]. Recent cohorts studying patients treated with recanalisation therapy have also shown similar results at three-month intervals, with mortality rates ranging from 35 to 41% and 90-day mRS scores of 0 to 3 between 36 and 44% [6,7,8,9].

The clinical presentation of BAO is diverse due to the large region of tissue supplied by the basilar artery [10]. Patients with BAO may exhibit symptoms of impaired consciousness, quadriparesis, and pupillary abnormalities, which have been associated with unfavourable outcomes [10,11]. Vertigo and hearing loss may act as warning signs of an impending vertebrobasilar ischemic stroke [12].

Atherosclerosis is a common aetiology of BAO, predominantly affecting the proximal and middle segments and accounting for approximately half of BAO cases [13]. Cardioembolism is another common aetiology of BAO, predominantly affecting the distal basilar segment and basilar tip. Underlying conditions such as vertebral artery dissection may also cause BAO [14]. Vertebral artery dissection occurs when a tear in the inner lining of the vertebral artery leads to the formation of blood clots and subsequent occlusion of the basilar artery [15]. Differentiating these etiologies may have implications for the appropriate treatment strategy and preventing recurrent events.

Recent randomised trials have demonstrated benefit for the use of endovascular therapy (EVT) in select patients with BAO [16,17]. Early recognition of BAO symptoms is crucial for initiating appropriate treatment, as delays in reperfusion therapy can significantly impact patient prognosis [9,18]. This review highlights current treatment options for BAO and the evidence supporting these treatments.

## 2. Methods

To summarise the current evidence on basilar artery occlusion management, a literature search was conducted using PubMed, covering articles from its inception through 30 April 2024. We focused our review on articles published in English. To narrow down the search, we combined terms including “thrombolysis”, “alteplase”, “rtPA”, “endovascular treatment”, “thrombectomy”, “stentretriever”, and “thromboaspiration”.

The study design of the included articles included case-control studies, cohort studies, comparative analyses, randomised trials, and meta-analyses. The retrieval process was further analysed by using filters such as “case-control study”, “cohort study”, “comparative analysis”, “randomized trial”, “meta-analysis”, “subgroup analysis”, and “pooled analysis”. These filters were used during the initial electronic search to ensure relevance and rigorous study design. The bibliographies of the identified articles were reviewed as a secondary strategy to find important studies that may have been missed in the primary search.

The study selection process was conducted in two stages. First, the titles and abstracts of the identified articles were screened to determine their relevance to the review. Then, the full texts of potentially relevant articles were retrieved and assessed for inclusion.

Data were extracted independently by one reviewer. Extracted data included study design, participant characteristics, interventions, outcomes, and key findings. Any discrepancies were resolved by consensus between the lead and senior author (DG, TNN).

### 2.1. Intravenous Thrombolysis

Management strategies for acute posterior circulation strokes, including acute reperfusion therapy, have historically been less emphasised in research compared to interventions for anterior circulation stroke [19,20,21]. IVT within 4.5 h of symptom onset is widely acknowledged as the standard treatment for acute ischemic stroke (AIS) of both the anterior and posterior circulation [22]. The Third International Stroke Trial conducted a subgroup analysis according to the location of the stroke. Although only 8% of the patients had posterior circulation strokes, no significant difference in clinical outcomes was found compared to anterior circulation stroke [23]. Similar results were observed in observational studies, where IVT was shown to have comparable efficacy in the posterior versus anterior circulations in a cohort of 5146 patients, of whom 753 (14.6%) had posterior circulation stroke [24]. At three months post-treatment, favourable mRS scores were reported in 45.2% of patients with posterior circulation stroke versus 37.5% with anterior circulation stroke, while the risk of symptomatic intracerebral haemorrhage (sICH) was nearly halved (relative risk: 0.49) [24]. A meta-analysis reinforced these findings by confirming lower rates of sICH for the posterior circulation stroke group compared to the anterior circulation stroke group [25]. In the extended time window up to 48 h from onset, a single-centre study of 184 patients showed that the use of IVT has been shown to produce good outcomes in half of patients in the absence of extensive baseline ischemia [26].

The location of BAO may impact the survival and treatment outcomes of patients receiving IVT. In a prospective registry, top-of-basilar clot location (*n* = 23) was associated with partial or complete recanalisation with an OR of 4.8 (95% CI 1.1–22, *p* = 0.048) compared to other occlusion sites [6]. This difference in terms of recanalisation rate may represent different underlying aetiologies [27] and/or the clot composition between top-of-basilar occlusions and other occlusion sites.

### 2.2. BAO Prospective Registries

In the Basilar Artery International Cooperation Study (BASICS) prospective registry, the outcomes of patients receiving IVT with or without intra-arterial therapy (IAT) and antithrombotic treatment were evaluated, wherein there was no clear superiority of either treatment strategy [3]. Patients with a mild-to-moderate deficit (*n* = 245) had a similar risk of poor outcome (mRS 4–5 or death) after IVT (adjusted RR 0.94, 95% CI 0.60–1.45) compared with antithrombotic treatment. In patients with severe strokes, there was a lower likelihood of poor outcomes with IVT (adjusted RR 0.88, 95% CI 0.76–1.01) or IAT (adjusted RR 0.94, 95% CI 0.86–1.02) compared to antithrombotic treatment [3]. Overall, the lack of superiority of either treatment group from the BASICS registry provided the rationale to proceed with a randomised trial for best medical management (BMM) versus EVT of BAO [28]. The findings with regard to benefit of EVT in more severe patients with BAO were also similarly shown in a state-wide prospective basilar registry in Germany [29].

Significant interest in endovascular therapy grew after the introduction of the stent retriever device in the 2015 endovascular randomised trials, predominantly including patients in the anterior circulation [30]. As recanalisation predicts better outcomes for BAO, there was a need for more study [7,31]. It wasn’t until 2020 that the first RCT using stent retriever-based devices for EVT in BAO was published, [32] followed by three more BAO RCTs [16,17,33].

Much of the evidence supporting EVT in BAO has relied on observational studies [3,34,35,36]. The BASICS international prospective registry did not demonstrate the effectiveness of EVT compared to IVT [3]. However, contrasting findings were observed in the BASILAR prospective registry, which included 829 BAO patients presenting within 24 h of the estimated onset of symptoms in China [36]. Among them, 647 patients received EVT and 182 received BMM. The BASILAR registry revealed improved ordinal mRS functional outcomes at 90 days (OR 3.08—95% CI 2.09–4.55) and higher mRS scores of 0–3 (OR 4.7—95% CI 2.53–8.75) in the EVT compared to BMM. Additionally, lower rates of 90-day mortality (47% vs. 70%) were noted in the EVT versus BMM groups despite an increase in sICH (7.1% vs. 0.7%) [36].

The Endovascular Therapy for Acute Basilar Artery Occlusion (ATTENTION) prospective registry in China, which included 1,672 BAO patients who underwent EVT (with IVT in 24%) and 462 patients treated with BMM (with IVT in 19%), confirmed similar findings as those shown in the BASILAR registry. The ATTENTION prospective registry showed more favourable functional outcomes (mRS 0–3: 40% vs. 29%, adjusted RR 1.42—95% CI 1.19–1.65) and reduced mortality rates (37% vs. 48%, RR 0.78, 95% CI 0.69–0.88) with EVT compared to BMM [35].

The ENDOSTROKE study, an international, investigator-driven, multicentre registry, included 148 patients who received EVT (with IVT in 59%) between 2011 and 2013. There were 34% with favourable outcomes, defined as mRS 0–2 at 90 days, 42% exhibited moderate clinical improvement, and the mortality rate was 35% [8].

### 2.3. Bridging Intravenous Thrombolysis with EVT

With regard to initiating IVT before EVT, a meta-analysis including two RCTs and 10 cohort studies showed that the EVT alone group had a lower rate of functional independence compared to the bridging therapy group (29% vs. 38%; RR 0.78, 95% CI 0.68–0.88, *p* < 0.001), lower independent ambulation (39% vs. 45%; RR 0.89, 95% CI 0.82–0.98, *p* = 0.01), and higher mortality (36% vs. 28%, RR 1.22, 95% CI 1.08–1.37, *p* = 0.001). However, no differences were detected in sICH between the two groups (6% vs. 4%; RR 1.12, 95% CI 0.74–1.71, *p* = 0.58) [37].

In a review of 110 patients who underwent IVT before thrombectomy, patients treated with Tenecteplase (TNK) had greater reperfusion rates compared to alteplase (tPA). Reperfusion of 50% or more of the index artery was observed in 26% of patients who received TNK, while only 7% of those who received tPA had the same level of reperfusion [38].

In summary, the combination of IVT plus EVT appeared to result in better functional independence, independent ambulation, and lower risk of mortality without increasing the incidence of sICH compared to EVT alone. However, given the non-randomised nature of these studies and the likely presence of confounders, further trials are needed to confirm these findings.

### 2.4. EVT Basilar Artery Occlusion Randomised Trials

Since 2020, four RCTs have examined the role of EVT in BAO (Table 1). The BEST and BASICS trials compared EVT to BMM [32,33]. Both RCTs failed to show evidence of the benefit of EVT over BMM in the treatment of BAO.

The BEST trial, published in 2020, assessed outcomes in patients with BAO presenting within 8 h of symptom onset across 28 centres in China. The primary outcome was a mRS score of 0–3 at 90 days, and the safety outcomes were sICH and mortality at 90 days. The trial was terminated after 131 patients were enrolled (66 in the EVT group and 65 in the BMM group) due to high crossover rates and poor recruitment (BMM to EVT crossover of 22%). IVT was administered in 27% of the BMM group and 32% of the EVT group. The intention-to-treat analysis showed comparable results for the primary outcome of mRS 0–3 at 90 days between the EVT and the BMM groups, with no significant difference (42% vs. 32%, OR: 1.74, 95% CI 0.81–3.74) [32]. All secondary outcomes (mRS 0–2, NIHSS and GCS after 24 h and 5–7 days) were comparable. Per-protocol and as-treated analyses showed that patients in the EVT group fared better for the primary outcome with mRS 0–3 at 90 days. The results were confounded by the loss of equipoise through the trial, leading to a reduced sample size (131 patients), lower than their target sample size of 344 patients [32].

The BASICS trial, published in 2021, was conducted at 23 centres in seven countries and enrolled patients within 6 hours of a stroke due to BAO [33]. The original inclusion criteria included patients aged <85 years old with NIHSS scores of ≥10, but due to slow enrollment, the criteria were expanded to include patients aged >85 years old and those with NIHSS scores of <10. The BASICS trial enrolled 300 patients, with 154 in the EVT group and 146 in the BMM group. IVT was used in 78.6% of the EVT group and in 79.5% of the BMM group, in contrast with the BEST trial, where IVT was used in a lower percentage of patients. In the intention-to-treat analysis, 66 of 154 patients (44.2%) in the EVT group had a favourable primary outcome of mRS score 0–3 at 90 days compared to 55 out of 146 patients (37.7%) in the BMM group, with no significant difference (Risk Ratio 1.18; 95% CI 0.92–1.50). Mortality at 90 days was 38.3% in the EVT group versus 43.2% in the BMM group (Risk Ratio 0.87, 95% CI 0.68–1.12) [33]. sICH occurred in 4.5% of patients in the EVT group compared to 0.7% in the BMM group. A subgroup analysis of patients with moderate deficits (NIHSS score 10–19) showed favourable outcomes for the EVT group compared to the BMM group, though the results are exploratory because of lack of power among the subgroups [33].

Recruitment was a challenge in both the BEST and BASICS trials [32,33]. There were 55% of patients in BEST and 29% of patients in BASICS who were treated outside the trial. Among patients with an NIHSS < 10, the BASICS study demonstrated benefit for medical management, in line with the BASICS and ATTENTION registries [3,35]. Despite the neutral results of the BEST and BASICS trials, an international survey of stroke and neurointerventional clinicians showed that most clinicians still believed in the efficacy of EVT [2,39,40]. Notwithstanding, the BEST and BASICS trials paved the way for patient selection in the subsequent generation of trials of EVT for BAO that highlighted consecutive enrollment and minimising crossover.

The ATTENTION RCT compared EVT to BMM in 340 patients presenting with BAO within 12 h of symptom onset. The study was conducted in 36 stroke centres across China from 2021 to 2022. Eligibility criteria included patients aged 18–80 years old. Given the data from the BASICS registry, [3] the BASICS trial, [33] and the ATTENTION registry results [35], which showed the treatment effect of EVT was related to the baseline NIHSS scores, the ATTENTION trial included only patients with a NIHSS score ≥ 10 [16]. ATTENTION used pc-ASPECTS [41,42] scoring to assess the extent of brainstem ischemia on NCCT, CTA, or DWI-MRI. Patients with a pc-ASPECTS score of <6 were excluded from the trial (indicating a larger territory of ischemia). There were 226 patients who received EVT (IVT in 31%), and 114 patients received best medical management (IVT in 34%). The EVT group showed a twofold increase in the primary endpoint of mRS 0–3 at 90 days compared to the BMM group (46% vs. 23% rate ratio: 2.06—95% CI (1.46–2.91)). Mortality rates were lower in the EVT compared to the BMM group (37% vs. 55%). sICH rates, according to the SITS-MOST criteria, at 24–72 h were 5% for EVT vs. 0% for BMM. These haemorrhage rates were consistent with the BEST and BASICS trials (4–8%) [16].

The BAO Chinese Endovascular Trial (BAOCHE) differed in patient selection [17]. It was conducted over a 5-year period in China with 217 patients with BAO who presented at 6–24 h after symptom onset. There were 110 patients who received EVT (IVT in 14%), and 107 patients received BMM (IVT in 21%). The trial was halted at interim analysis because of meeting the primary endpoint with superiority of EVT. The inclusion criteria of the trial were more permissive than ATTENTION, enrolling patients with an NIHSS score ≥ 6, along with a pc-ASPECTS score ≥ 6 and a pons-midbrain index score ≤ 2 (higher score indicating a presence of large infarct) [43]. The primary outcome of mRS 0–3 at 90 days was achieved in 46% of patients in the EVT group and 24% in the control group (RR: 1.81; 95% CI (1.26–2.6)). The sICH rates were higher in the EVT group (6%) compared to the control group (1%). Mortality at 90 days was 31% in the EVT group vs. 42% in the control group (RR: 0.75; 95% CI: (0.54–1.04)) [17].

A systematic review and meta-analysis of the four RCTs that included 988 patients found that the primary endpoint of a favourable functional outcome (mRS 0–3) at 90 days was achieved in 251 of 556 patients (45.1%, 95% CI 41–49.3%) in the EVT group compared to 128 of 432 patients (29.6%, 95% CI 21.7–36.4%) in the BMM group [4]. Despite an increase in sICH cases among the EVT group, with rates of 5.4% (95% CI 3.5–7.2%) compared to those in the BMM group at 0.8% (95% CI 0.0–2%), individuals receiving EVT had reduced mortality when compared with the BMM group. In a subgroup analysis of 78 BAO patients with an NIHSS < 10, the frequency of favourable or excellent outcomes at 90 days, as well as mortality, was similar in the EVT as compared to the BMM group, while the frequency of sICH was 8.1% in the EVT group compared to none in the BMM group [4].

The success of ATTENTION and BAOCHE, in contrast to BEST and BASICS, might be explained by patient selection and consecutive enrollment. New trials used inclusion criteria and infarct volume scales, while the preceding two randomised controlled trials did not include a quantitative evaluation of ischemic damage.

The reason for positive results in the ATTENTION and BAOCHE trials compared to the neutral results of the BEST and BASICS trials may be attributed to poorer outcomes in the BMM group, owing to a lower rate of IVT use. As the treatment windows of the ATTENTION and BAOCHE trials were extended to 12 and 24 h, respectively, it is expected that there would be lower rates of IVT administration as more patients present outside the 4.5 h time window for IVT eligibility [44]. In a subgroup analysis of ATTENTION and BAOCHE, where BMM included 100% IVT, no superiority of EVT was observed [16]. The risk of sICH was also lower after IVT, which is reassuring. Given the potential of IVT, considerations for use beyond 4.5 h should be explored in future research. Ongoing trials are testing the efficacy of IVT in patients with BAO in the late time window (POST-ETERNAL, NCT05105633, ATTENTION-IV LATE, NCT05701956).

The generalisability of the results of these trials to other patient populations and healthcare settings remains uncertain [45]. Both the positive trials were conducted in China, where rates of intracranial atherosclerotic disease (ICAD) are significantly higher [46]. ICAD is more common in the posterior circulation than in the anterior circulation and may result in poorer clinical outcomes than those resulting from embolic occlusions [47]. Patients with ICAD were also found to have a lower rate of favourable outcomes compared to patients with embolic occlusion of 10.5% vs. 37.5, respectively, in one study [47]. The efficacy of BMM might be affected in such trials, as IVT performs better in distal occlusions due to embolism [6,48]. ICAD was the cause of stroke in nearly 44% of patients in the ATTENTION trial and 66% in the BAOCHE trial, compared to 52% in BEST and 35% in BASICS [4].

### 2.5. Angioplasty and Stenting for ICAD-Related BAO

Intracranial or extracranial angioplasty and stenting were used at a higher rate in the BAOCHE and ATTENTION trials, likely due to the increased prevalence of atherosclerotic disease [16,17]. Angioplasty and stenting could be considered for posterior circulation LVOs in scenarios where there is persistent residual severe stenosis or re-occlusion, particularly in situations of insufficient restoration of blood flow or a heightened risk of re-obstruction [49,50]. However, the basilar artery presents challenges due to its smaller diameter and perforator arteries, increasing the potential for complications during balloon angioplasty or stent placement [51]. Prompt stenting intervention is often necessary for intracranial atherosclerosis-related large vessel occlusion (ICAS-LVO) in the posterior circulation [52,53,54].

Comparing the use of intracranial angioplasty and stenting, in the ATTENTION trial, it was used in 40% of patients (88/221) compared to 17% in BASICS (26/154). In BAOCHE, 55% of patients were treated with angioplasty and stenting. Recurrent narrowing in ICAS-LVO occurs frequently and is likely attributed to platelet activation, significant residual stenosis, or both [46]. Therefore, urgent intervention should prioritise inhibiting platelet activity and maintaining vessel patency. Several previous reports have recommended the use of a glycoprotein IIb-IIIa inhibitor (GPI) for platelet inhibition [55]. Intra-arterial or intravenous infusion of low-dose GPI (Tirofiban, 0.5–1.5 mg or Reopro, 3–10 mg) was effective for resolution or prevention of re-occlusion of ICAS-related LVO [56,57]. Moreover, in a subgroup analysis of the RESCUE BT randomised trial, patients with anterior circulation ICAD-LVO had better 90-day functional outcomes (mRS 0–2) if they received preceding intravenous tirofiban compared to placebo (adjusted OR 1.68—95% CI 1.11–2.56, *p* = 0.02) [58,59].

In the ANGEL-ACT prospective registry of 93 patients with acute BAO who failed EVT, 81 (87.1%) received rescue therapy, with a 92.6% recanalisation rate. Compared with patients who did not receive rescue therapy (*n* = 12), the rescue therapy group had a significantly higher rate of favourable outcomes at 90 days (51.9% vs. 16.7%, *p* = 0.023) [52]. However, in the overall ANGEL ACT registry, including patients with anterior circulation LVO, the benefit of rescue angioplasty and/or stenting was not observed in patients who had residual severe stenosis but substantial reperfusion [60]. Therefore, rescue therapy should be considered in patients with underlying atherosclerotic stenosis and failed reperfusion (e.g., mTICI 0-2a) to improve outcomes after EVT.

### 2.6. Basilar Artery Occlusion Thrombectomy Technique

The choice of initial thrombectomy technique may also be influenced by the presumed cause of BAO. The most commonly utilised mechanical thrombectomy techniques are stentretriever, contact aspiration, or their use in combination. While both the ATTENTION and BAOCHE trials showed high rates of successful reperfusion, the latter predominantly utilising stentretriever techniques, the optimal technical and anaesthesia approach remains unknown [53,61].

For anterior circulation stroke, the ASTER and COMPASS RCTs revealed comparable outcomes for first-line stentretriever and contact aspiration [62,63]. Nevertheless, there is a lack of clarity regarding their impact on posterior circulation stroke [53,64]. A RCT comparing stentretriever and contact aspiration for patients with acute BAO is ongoing (NCT05320263; PC-ASTER). Stentretriever might be the favoured option for the primary recanalisation of an ICAD-related LVO rather than aspiration [65,66]. According to a 2019 study, the rate of switching to an alternative thrombectomy after failure of the front-line technique was significantly higher in the contact aspiration than in the stentretriever group (40% vs. 4.3%; OR 2.543, 95% CI 1.893–3.417), which was also demonstrated in a 2024 multicentre BAO study in the United States (22% vs. 6%, *p* < 0.01) [67,68]. First-line contact aspiration may have some advantages in treating embolic BAOs. In a retrospective study, first-line aspiration was associated with a shorter procedure time, better reperfusion, and better clinical outcome than stent retriever thrombectomy in patients with ischemic stroke based on large vessel occlusion in the posterior circulation [69]. In a meta-analysis of 11 studies and 1014 patients, there were higher odds of successful recanalisation with first-line aspiration compared to first-line stentretriever (OR 1.64, 95% CI 1.10–2.45) [70].

In the After the BEST of BASICS (ABBA) survey, which included 543 interventionists from 52 countries, contact aspiration was selected as the primary method for embolic aetiology by 50.3% of respondents, while a combination of stentretriever and aspiration was favoured for ICAD occlusion by 40.5% [53]. Adjunctive antithrombotic therapy in BAO lacks sufficient data to inform treatment decisions. A study published in 2020 compared tirofiban and oral antiplatelet therapy to oral antiplatelets in patients undergoing EVT for vertebrobasilar occlusion. Among the 105 patients, 74 underwent EVT + tirofiban + oral antiplatelet therapy, while 31 underwent EVT + oral antiplatelet drug therapy. EVT + tirofiban + oral antiplatelet therapy resulted in higher recanalisation rates compared to EVT + oral antiplatelet drug therapy (93.24% vs. 77.42%; *p* = 0.038), while there was no variation in the risk for sICH, 90-day mortality, and functional independence outcomes between the groups [71].

### 2.7. Other Considerations: Sex Differences in BAO, Mild Deficits

Across the four BAO RCTs, it is noteworthy that over two-thirds of the participants were male [4]. It is unclear whether this disparity in gender representation indicates challenges in recruiting female participants for RCTs, a lower incidence of BAO in females, or proper assessment of female eligibility for EVT. However, in China, it could indicate sex-based differences in developing LVO and its associated vascular risk factors. Findings from the ANGEL-ACT LVO registry and ANGEL-REBOOT RCT in China showed a higher proportion of males with ICAD-related LVO, [72] possibly impacted by a notably higher prevalence of smoking among men compared to women in China [73,74]. This highlights an opportunity for intensive smoking cessation counselling targeting men as part of stroke prevention efforts within this population.

A salient question lingers within the clinical community: Does EVT confer a tangible benefit for BAO patients presenting with minor neurological impairment? The BAOCHE trial broadened its inclusion criteria to include patients with NIHSS scores as low as 6, yet the representation of individuals within the mild deficit bracket (NIHSS scores between 6 and 9) remains scarce. This paucity of data, coupled with insights from the BASICS trial and the ATTENTION and BASIC registries, indicates a lack of definitive evidence supporting recommendations for the potential benefit of EVT for patients harbouring NIHSS scores below 10 [45,75].

### 2.8. Imaging Considerations

Non-contrast CT (NCCT), along with CT perfusion, has been utilised to detect AIS patients with salvageable tissue for EVT. In patients with favourable ASPECTS, selection by NCCT alone has shown comparable outcomes with advanced imaging in anterior LVO patients, even when presenting in later windows [76,77]. However, the diagnostic effectiveness of CTP and NCCT is constrained in posterior circulation stroke because of the impact of blood flow speed and bony structure in the posterior fossa [78]. Diffusion-weighted MRI is more sensitive to delineate infarcted tissue but has a lower utilisation in the emergency department due to availability and a longer scanning time [79]. Imaging the ischemic tissue and collateral patterns could assist in identifying patients who are most likely to benefit or be harmed from treatment. The pc-ASPECTS (posterior circulation-Alberta Stroke Program Early CT Score) estimates severe tissue hypoperfusion with non-viability and can be assessed on NCCT, CT-angiography source images, or DWI [41]. pc-ASPECTS on CT angiography source images independently predicted death and functional independence at 1 month in the CT angiography subgroup of patients in the BASICS registry [80]. BAOCHE detailed their approach, wherein DWI or CTA source imaging [CTA-SI] were suggested as the favoured imaging method. The pc-ASPECTS and pons-midbrain index were reported on CTA-SI in 51.2% of patients, DWI in 31.8%, and NCCT in 15.2% [17]. In a study of 130 patients with suspected vertebrobasilar ischemia, the sensitivity for ischemic changes was improved with CTASI compared to NCCT (65% [95% CI, 57% to 73%] versus 46% [95% CI, 37% to 55%], respectively). The pc-ASPECTS score on CTASI but not NCCT predicted functional independence (OR 1.58; *p* = 0.005 versus 1.22; *p* = 0.42, respectively) [41]. Of note, there was no difference in outcome detected in relation to the pc-ASPECTS in the subgroup analysis of the BASICS prospective registry. This may be partially attributed to the assessment of pc-ASPECTS on non-enhanced CT images in 54% of participants (129 in the endovascular therapy group and 115 in the medical therapy group) and the recruitment of a substantial number of individuals with a mild impairment (NIHSS score < 10) [80].

## 3. Conclusions

The evolving paradigm of BAO management has been driven by the therapeutic potential of endovascular therapy. As clinical trials have broadened the horizons of EVT, they also underscore the potential of IVT, necessitating further exploration into its utility, especially in expanded time windows [9,26]. The search to identify the optimal first-line technique in BAO intervention persists, as the landscape is further complicated by the underlying aetiology—whether ICAD or embolic. Beyond the basilar artery, understanding the optimal management, whether medical or endovascular, for posterior cerebral artery occlusion is an area of ongoing study [64,81,82,83]. Adjuvant intra-arterial thrombolysis administered after successful EVT is also undergoing study [84,85]. Through continued research collaborations, the stroke community aspires to mitigate the impact of BAO, thereby enhancing patient outcomes in both the acute and extended spectrums of care.

## Figures and Tables

**Table 1:** Characteristics of basilar artery occlusion randomised trials.

Trial Summary	BEST	BASICS	ATTENTION	BAOCHE
Years trial conducted	2015–2017	2011–2019	2021–2022	2016–2021
No. of patients enrolled/target sample size	131/344 (66 EVT, 65 MM)	300/300 (154 EVT, 146 MM)	342/342 (228 EVT, 114 MM)	217/318 (110 EVT, 107 MM)
Countries	China	7 Countries	China	China
Estimated symptom onset to randomisation (h)	0–8	0–6	0–12	6–24 *
Age inclusion criteria, years (yr)	≥18	Initially18–85 (extended later to ≥18)	≥18	18–80
NIHSS criteria	Not specified	Initially ≥10 (extended later to NIHSS < 10)	≥10	Initially ≥10 (extended to NIHSS ≥ 6
Pre-stroke mRS	0–2	0–2	≤80 years: 0–2	≤1
			>80 years: 0	
Imaging selection criteria	No evidence of intracranial haemorrhage, significant cerebellar mass effect, acute hydrocephalus, or extensive bilateral brainstem ischemia on CT or MRI	No evidence of intracranial haemorrhage, extensive bilateral brainstem infarction on CT, cerebellar mass effect, or acute hydrocephalus on neuroimaging	NCCT/CTA-SI/DWI pc-ASPECTS	pc-ASPECTS ≥ 6 or pons-midbrain index of ≤2
Endovascular treatment modality	At the discretion of treating physician	At the discretion of treating physician	At the discretion of treating physician	Solitaire stentretriever
Crossover				
EVT to MM	3/66, 5%	3/154, 1.9%	3/226, 1.3%	1/110, 0.9%
MM to EVT	14/65, 22%	7/146, 4.8%	3/114, 2.6%	4/107, 3.7%
Trial demographics				
Age (yr)				
EVT	62 (50–74)	66.8 ± 13.1	66.0 ± 11.1	64.2 ± 9.6
MM	68 (57–74)	67.2 ± 11.9	67.3 ± 10.2	63.7 ± 9.8
Male sex				
EVT (%)	73	64.9	66	73
MM (%)	80	65.8	72	74
Baseline NIHSS				
EVT	32 (18–38)	21	24 (15–35)	20 (15–29)
MM	26 (13–37)	22	24 (14–35)	19 (12–30)
Baseline pc-ASPECTS				
EVT	8 (7–9)	10 (8–10)	9 (8–10)	8 (7–10)
MM	8 (7–9)	10 (8–10)	10 (8–10)	8 (7–10)
IVT				
EVT	18 (27)	121 (78.6)	69 (31)	15 (13.6)
MM	21 (32)	116 (79.5)	39 (34)	23 (21.5)
ICAD ^†^				
EVT	37 (56)	53/146 (36.3)	90/226 (40)	N/A
MM	32 (49)	43/132 (32.6)	33/114 (29)	N/A

Demographic data are median (IQR), *n* (%), or mean ± SD unless otherwise indicated. BEST, Basilar Artery Occlusion Endovascular Intervention versus Standard Medical Treatment; BASICS, Basilar Artery International Cooperation Study; ATTENTION, Endovascular Treatment for Acute Basilar Artery Occlusion: A Multicentre Randomised Clinical Trial; BAOCHE, Basilar Artery Occlusion Chinese Endovascular Trial; EVT, endovascular thrombectomy; MM, medical management; NIHSS, National Institutes of Health Stroke Scale; mRS, modified Rankin Scale; CT, computed tomography; MRI, magnetic resonance imaging; NCCT, non-contrast CT; CTA, computed tomography angiography; SI, source images; DWI, diffusion-weighted imaging; pc-ASPECTS, posterior circulation Acute Stroke Prognosis Early CT Score; IVT, intravenous thrombolysis; ICAD, intracranial atherosclerotic disease; N/A, not available; IQR, interquartile range. * Time from symptom onset; ^†^ The percentage of patients with intracranial atherosclerotic disease was not reported in the BAOCHE publication. Note that 41/110 (37.3%) underwent intracranial stenting in the endovascular group. Large artery atherosclerosis was reported as the cause of stroke in 75/110 (68.2%) in the EVT group and in 69/107 (64.5%) in the control group in the BAOCHE study. Reference: Abdalkader M, et al. [4].
